# Associations of hair cortisol levels with violence, poor mental health, and harmful alcohol and other substance use among female sex workers in Nairobi, Kenya

**DOI:** 10.1007/s44192-024-00086-1

**Published:** 2024-08-28

**Authors:** Mamtuti Panneh, Qingming Ding, Rhoda Kabuti, The Maisha Fiti study champions, John Bradley, Polly Ngurukiri, Mary Kungu, Tanya Abramsky, James Pollock, Alicja Beksinska, Pooja Shah, Erastus Irungu, Mitzy Gafos, Janet Seeley, Helen A. Weiss, Abdelbaset A. Elzagallaai, Michael J. Rieder, Rupert Kaul, Joshua Kimani, Tara Beattie

**Affiliations:** 1 Department for Global Health and Development, London School of Hygiene & Tropical Medicine, London, UK; 2 Schulich School of Medicine and Dentistry, Robarts Research Institute, Western University, London, ON Canada; 3 Partners for Health and Development in Africa, Nairobi, Kenya; 4 MRC International Statistics and Epidemiology Group, Department for Infectious Disease Epidemiology, LSHTM, London, UK; 5 Department of Immunology, University of Toronto, Toronto, Canada; 6 Department of Medicine, University of Toronto, Toronto, Canada

## Abstract

Violence, poor mental health, and harmful substance use are commonly experienced by female sex workers (FSWs) in sub-Saharan Africa, all of which are associated with increased HIV susceptibility. We aimed to investigate the associations between violence, poor mental health and harmful alcohol/substance use with hair cortisol concentration (HCC) levels as a potential biological pathway linking the experiences of these stressors and HIV vulnerability. We used the baseline data of the *Maisha Fiti* study of FSWs in Nairobi, Kenya. Participants reported recent violence, poor mental health, and harmful alcohol/substance use. Hair samples proximal to the scalp were collected to measure cortisol levels determined by ELISA. We analysed the data of 425 HIV-negative respondents who provided at least 2 cm of hair sample. The prevalence of recent violence was 89.3% (physical 54.6%; sexual 49.4%; emotional 77.0% and financial 66.5%), and 29.1% had been arrested due to sex work. 23.7% of participants reported moderate/severe depression, 11.6% moderate/severe anxiety, 13.5% PTSD and 10.8% recent suicidal thoughts and/or attempts. About half of the participants (48.8%) reported recent harmful alcohol and/or other substance use. In multivariable linear regression analyses, both physical and/or sexual violence (adjusted geometric mean ratio (aGMR) = 1.28; 95% CI 1.01–1.62) and harmful alcohol and/or other substance use (aGMR = 1.31; 95% CI 1.03–1.65) were positively and independently associated with increased HCC levels. Findings suggest a role of violence and substance use in elevated HCC levels, which could increase HIV risk due to cortisol-related T cell activation. However, longitudinal and mechanistic studies are needed to confirm this hypothesis.

**Supplementary Information:**

The online version contains supplementary material available at 10.1007/s44192-024-00086-1.

## Introduction

The prevalence of lifetime intimate partner violence (IPV) among women of reproductive age (aged 15–49 years) in sub-Saharan Africa (SSA) was 33% in 2018, compared to 27% globally [1]. Female sex workers (FSWs) in SSA face a higher risk of violence compared to the general population of women, attributed to factors such as the criminalisation of sex work, as well as stigma and discrimination [2]. They are also at increased risk of developing poor mental health [3] and disproportionately engage in harmful substance use, including alcohol, often as a coping mechanism to deal with the daily challenges of sex work [4]. In addition, FSWs are at an increased risk of acquiring HIV infection and other health problems compared to women in the general population [3, 5]. For example, a report in 2019 revealed that globally, FSWs were 30 times more likely to be diagnosed with HIV compared to the general female population of women of reproductive age [6].

Similar to other parts of the world, FSWs in Kenya are at a high risk of experiencing violence, poor mental health and harmful substance use [7–9]. Recent baseline findings from the *Maisha Fiti* study in Nairobi, which provided data for this study, revealed that approximately 81% experienced violence (physical/sexual/emotional) from intimate and non-intimate partners in the past six months [9]. Almost half (49%) reported symptoms of mild, moderate/severe depression, 30% engaged in harmful alcohol use, and 31% had harmful levels of use of other illegal substances such as cannabis, cocaine, and hallucinogens [9]. The prevalence of HIV among FSWs in Nairobi is approximately 25%, estimated to be five times higher than the general population of Kenyan women [10, 11]. Although sex work in Kenya is not explicitly criminalised by federal law, municipal by-laws, such as in Nairobi County, may prohibit it [12]. This punitive quasi-criminalized nature of sex work creates an atmosphere that increases FSWs’ risks of violence from various perpetrators, including law enforcement officers who unlawfully arrest and assault them for selling sex [12, 13].

There is strong evidence that violence, poor mental health, and harmful substance use can be bidirectional associated with each other [14–16]. They are also known to be strongly associated with increased HIV infection [17–20]. However, the link between these exposures, especially violence and increased HIV acquisition, may not be entirely explained by behavioural risk pathways and is not fully understood [21]. For example, a systematic review of epidemiological studies in SSA found strong evidence of an association between HIV and physical violence and emotional violence, respectively, but no evidence of an association between HIV and sexual violence [22]. This suggests that the association between HIV and violence at a broader societal level cannot be solely attributed to HIV transmission during instances of sexual violence [22]. In addition, some studies have shown that psychological stress and physical abuse (which includes violence) may induce immune activation [23, 24]. There are physiological reasons that violence, poor mental health and harmful alcohol or other substance use may increase HIV risk, for example, by activating the physiological stress response system, which can result in a range of complex bi-directional interactions between the endocrine, nervous and immune systems [21]. One of the main neuroendocrine systems in the stress response system is the hypothalamic–pituitary–adrenal (HPA) axis, which releases the hormone cortisol when activated. Cortisol, often called the "stress hormone," can indicate HPA activation in the brain [25]. It plays a crucial role in regulating most vegetative functions and is known to suppress the immune system during a physiological stress response to prevent damage to the body [21]. However, dysregulated cortisol levels due to chronic stress are known to exact pathogenic effects in the development of many conditions, including chronic inflammation [26]. For example, research suggests increased cortisol levels may fail to downregulate the inflammatory responses to viruses and other triggers [27]. Additionally, cortisol is associated with key markers of T-cell activation (e.g., CD8 + T-cells), which have been reported to be associated with HIV susceptibility and progression [28, 29]. According to Patterson et al., the link between chronic psychological stress and impaired immune regulatory responses to anti-inflammatory signals may be due to chronic stress dysregulating the HPA axis. This reduces immune cell sensitivity to cortisol, impairs inflammatory regulation, and leads to higher T-cell activation [28].

Cortisol has often been measured using blood, saliva, or urine. These measure short-term cortisol levels only, which makes understanding the relationship between long-term stress exposure and the development of stress-related health problems challenging [30]. In contrast, the analysis of cortisol in hair (scalp hair) is a reliable long-term cortisol measurement, providing a retrospective reflection of cortisol levels over several months [30, 31]. Evidence has shown dysregulated cortisol levels in adults without psychiatric disorders who have been exposed to adverse stressful environments [32]. However, research on the relationship between violence and cortisol and cortisol and mental health, respectively, have produced mixed results [33, 34]. A systematic review of hair cortisol and mental health problems generally found an increase and a decrease in cortisol levels in individuals with major depression and anxiety (generalised anxiety disorder, panic disorder), respectively [34]. Also, there is evidence that the dysregulation of the HPA axis is linked with excessive alcohol consumption; active drinkers are known to usually have elevated cortisol levels, which gradually decrease with abstinence [35]. In contrast, a few studies have reported lower cortisol levels or insignificant findings between alcohol-dependent participants compared with healthy controls [36]. These discrepancies have been linked to the severity, timing, and type of stressors experienced as different adverse events are known to uniquely impact the HPA axis response to stress. Research has indicated that repeated or prolonged stress may initially lead to increased cortisol levels (hypercortisolism) during the beginning and early post-stress phases. However, this may transition to reduced cortisol levels (hypocortisolism) [37, 38].

FSWs’ vulnerabilities to various stressors and poor physical health, including HIV infection, calls for a better understanding of how these stressors are associated with cortisol levels in this population. To our knowledge, only one study globally has previously examined cortisol levels among FSWs, which was conducted in Mombasa, Kenya [39]. In that cross-sectional study with a sample size of 283 FSWs, participants who were recently (in the past 12 months) exposed to gender-based violence (physical/sexual/emotional) had higher cortisol levels than their unexposed counterparts [39]. Using a larger sample size of 425 FSWs in Nairobi, Kenya, the aim of this current study is to examine whether recent violence of different forms, poor mental health and harmful alcohol or substance use are independently associated with hair cortisol levels. Given the evidence indicating that repeated stress may elevate cortisol levels at the beginning and early post-stress phases [37, 38], we hypothesised that recent experiences of violence, poor mental health, and harmful substance use may be associated with increased levels of hair cortisol. Evidence suggests that cortisol communicates with the immune system and that cortisol and systemic inflammation may be bi-directionally associated [21]. Thus, we also examined if hair cortisol levels were associated with C-reactive Protein (CRP) levels—an acute systemic inflammatory marker. Understanding the physiological relationships between violence, poor mental health, and harmful alcohol/substance use with cortisol levels, as well as between cortisol levels and systemic inflammation, is vital in determining how these exposures may increase HIV risk.

## Methods

### Study design and recruitment

This study was cross-sectional, using baseline behavioural and biological survey data collected from the *Maisha Fiti* study from June to December 2019. The design of the *Maisha Fiti* study was in collaboration with the FSW community in Nairobi and the staff and peer educators of the Sex Work Outreach Program (SWOP) clinics in Nairobi. Approximately 73% (29,000) of FSWs in Nairobi County were registered at one of seven SWOP clinics across Nairobi. Each FSW attending a SWOP clinic is assigned a unique barcode for identification. FSWs who had visited any SWOP clinic in the previous 12 months, indicating active engagement in sex work, and who met the eligibility criteria below were randomly selected to participate in the *Maisha Fiti* study.

The eligibility criteria for the sampling frame were as follows: (i) aged 18–45 years, (ii) attended a SWOP clinic in the past 12 months, and (iii) no chronic illness (excluding HIV) such as diabetes, rheumatoid arthritis, asthma, and TB infection that could impact the immune system. A total of 10,292 of the 29,000 FSWs met these criteria and were included in the sampling frame. The desired sample size was 1000 FSWs; however, 1200 FSWs were selected to allow for non-response and non-eligibility since additional exclusion criteria (assessed during study enrolment) were current pregnancy (urine samples were tested for pregnancy) and breastfeeding. The number of FSWs selected per clinic was proportional to clinic size. Women aged less than 25 years were oversampled to allow adequate power for analyses stratified by age. Selected women were telephoned and informed about the study (in English/Swahili). Those interested were invited to visit the study clinic, where they received detailed information about the study. Participation was voluntary; interested women were screened for eligibility, and those who met the criteria provided written informed consent. Details about the sample size calculation and selection of participants can be found elsewhere [9, 11]. This current paper focused on HIV-negative participants.

### Data collection process

#### Behavioural-biological surveys:

The behavioural survey captured data on socio-demographics, adverse childhood experiences (ACEs), sexual practices and behaviours, stigma, social support, violence experiences, mental health problems, and harmful substance use. After women responded to the behavioural survey, biological samples, including urine, blood, vaginal swabs, and hair samples, were collected for biological tests. Urine samples were provided to test for gonorrhoea and chlamydia infection using GeneXpert Assay. Blood samples were used to test for HIV, syphilis and CRP levels using rapid HIV tests, rapid plasma regain assay and Nano checker reader, respectively. Positive HIV results were confirmed using HIV DNA GeneXpert. Vaginal swabs (self-collected) were used to test for bacterial vaginosis (BV; Gram’s stain and Nugent scoring) and *Trichomonas vaginalis* (TV; OSOM Trichomonas Rapid Test; SEKISUI Diagnostics, LLC). Hair samples were used to test for cortisol levels, as described below.

### Study variables

#### Outcome variable

The main outcome variable was *hair cortisol concentration (HCC)*. For each participant, about 50 hair strands were cut by a research team member from the posterior vertex next to the scalp using clean scissors. The scalp end of the hair sample was labelled, and the sample was packaged in aluminium foil and stored in a cool, dry location for storage. Of the 1003 women recruited to participate in the *Maisha Fiti* study, 746 were HIV-negative at baseline [9], of whom 736 (98.7%) provided hair samples. Several of the women had either extremely short hair or hair samples with < 50 hair strands. To allow for sufficient samples to proceed with hair cortisol testing assays, we excluded samples less than 2 cm long (a hair length of about 6 cm was ideal), leaving 425 women with viable hair samples. The proximal 2 cm of hair from the scalp was analysed for cortisol concentration and represented cumulative cortisol concentration in the 2.5 months prior to the survey based on the average African hair growth rate of 0.79 cm per month [39]. Hair cortisol was analysed using an established ELISA technique [40] and expressed as nanogram/gram (ng/g) of hair mass.

#### Main exposure variables

The three main categories of exposures were: (i) experience of recent violence of different forms, (ii) mental health problems, and (iii) harmful substance use.

*Violence of different forms:* We asked participants about their experiences of physical, sexual and emotional violence in the past six months from both intimate partners and non-IPs (e.g., clients, police, strangers, etc.). The WHO Violence Against Women 13-item questionnaire, which assesses the frequency and severity of violence (physical, sexual, and emotional) perpetrated by intimate partner violence (IPV) in the past 12 months, was adapted to include violence perpetrated by non-intimate partners by repeating the IP questions to ask about non-IPs [41]. We inquired about experiences of violence ever and in the past six months. For questions assessing physical violence, women who responded "yes" to any of the questions were coded as "yes"; the same approach was used to code for emotional and sexual violence. Financial/economic violence, as used elsewhere, [42] was assessed by asking respondents if, in the past six months, a client refused to pay or had to be forced to pay after sex (yes/no). Each form of violence was categorised as binary (yes/no), irrespective of whether they had experienced other forms of violence. This means that, for example, women in the unexposed category for physical violence may nevertheless have been exposed to other types of violence, such as emotional violence. Women who experience physical and/or sexual violence tend to have multiple health implications, including increased HIV infection [43]. This definition of violence has frequently been used in other cohorts of FSWs [14, 44]. Thus, we created a combined variable for experiencing physical and/or sexual violence in the past six months. In a separate question, we also asked respondents if they had been recently arrested by police for selling sex with a binary response (Yes/No) (*Have you been arrested in the past six months because you were a sex worker?).*

*Mental health problems*: Mental health problems were measured using validated tools with high reliability and validity in Kenya. The Patient Health Questionnaire (PHQ-9) was used to measure depression symptoms (score ≥ 15 = moderate/severe depressive disorder) [45] in the past two weeks; The Generalized Anxiety Disorder (GAD-7) questionnaire for anxiety (score ≥ 10 = moderate/severe anxiety)[46] in the past two weeks and the Harvard Trauma Questionnaire (HTQ-17) for PTSD (score ≥ 2 positive for PTSD) [47] in the past month. These tools are all based on the Diagnostic and Statistical Manual of Mental Disorders (DSM-1 V) [48].

Suicide risk was assessed by a two-item questionnaire, which included recent suicidal thoughts (‘having thoughts about ending your life’) and recent suicide attempts (‘*having attempted to end your life’*) in the last 30 days. Due to the small number of women reporting a recent suicidal attempt and the overlap between a recent suicide attempt and thoughts, these two items were combined into a binary variable (recent suicidal thoughts and/or recent suicidal attempts). Research has shown that previous suicidal thoughts and/or suicide attempts increase the lifetime risk of further suicide attempts and completed suicide [49, 50].

*Harmful substance use:* The WHO ASSIST (Alcohol, Smoking and Substance Involvement Screening Test) tool was used to assess alcohol risk (cut-off scores: low risk 0–10; moderate risk 11–26; high risk 27 +) and other substance use risk (cannabis, cocaine, amphetamines, hallucinogens, sedatives and inhalant) in the last three months (cut-off scores: low risk 0–3; moderate risk 4–26; high risk 27 +) [51]. After initial exploratory analyses showed that harmful alcohol use (moderate/severe) and other substance use problems (moderate/severe) were similar with regard to HCC levels, these two measures were combined into a dichotomous variable (moderate/severe alcohol and/or other substance use problem). Tobacco use was assessed separately with a binary response for use in the past three months (Yes/No).

#### Conceptual framework

**Fig. 1:**
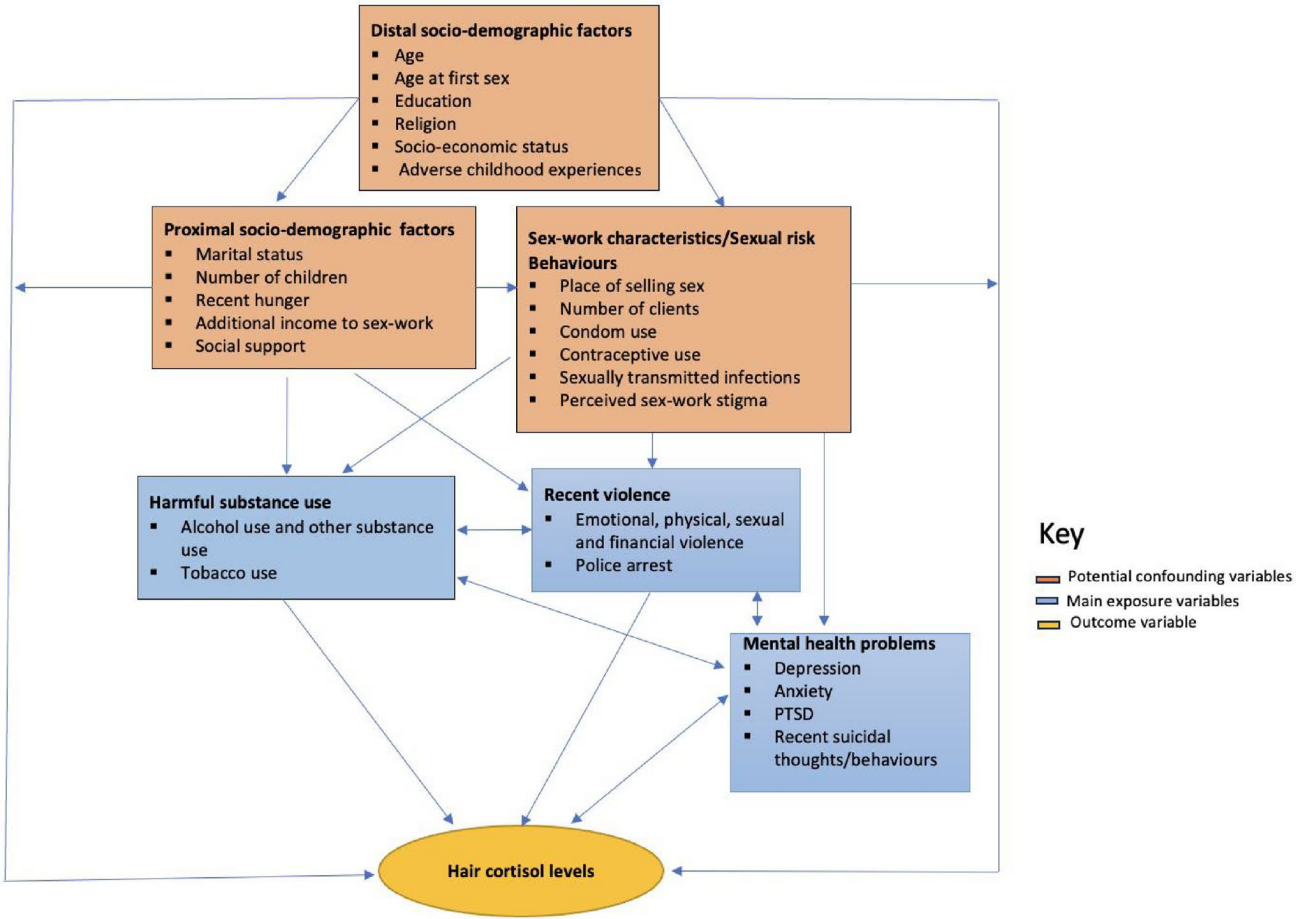
A conceptual framework illustrating the potential risk factors influencing hair cortisol levels among female sex workers in Nairobi, Kenya

Drawing on the literature, our experiences in the field, and the eco-social life course theory [52], we developed a conceptual framework to guide our analyses (Fig. 1). The eco-social life-course framework theorises how societal and ecological context exposures can be biologically embodied, causing health and disease disparities [52]. In Fig. 1, the rectangles in blue are the main exposure variables of interest, while the yellow sphere is the main outcome of interest (hair cortisol levels). Orange rectangles represented variables upstream of both the exposure and outcome variables and were therefore considered potential confounders. Variable definitions for potential confounders (orange rectangles) are explained in Table 1.

**Table 1:** Definition of some covariates used in the study

Variables	Question/measurement tool	Category
Childhood and distal Socio-demographic factors		
ACEs	WHO Adverse Childhood Experiences International Questionnaire (ACE-IQ). The total number of Adverse Childhood Experiences was defined as answering yes to: When you were growing up, during your first 18 years of life, (i) Did you live with a household member who was a problem drinker or alcoholic, or misused street or prescription drugs? (ii) Did you live with a household member who was depressed, mentally ill or suicidal? (iii) Did you live with a household member who was ever sent to jail or prison? (iv) Were your parents ever separated or divorced? (v) Did your mother, father or guardian die? (vi) Did you witness violence in the home (ACE 4.6, 4.7, 4.8) (vii) Did you experience emotional violence (ACE 5.1, 5.2) (viii) Did you experience physical violence (ACE 5.3, 5.4) (ix) Did you experience sexual violence (ACE 5.5, 5.6, 5.7, 5.8) (x) Did you experience community violence (ACE 7.1–7.3) (xi) (ACE 8.1–8.4) (xii) Did you ever live on the streets? [8]	Ordered categorical variable (each ACE scores one point): < 4, 5–8, 9–12 [8]
Age at first sex	How old were you when you first had penile insertive vaginal sex with a male partner?	< / = 15, 16–17, 18 +
Socio-economic status (SES)	14 household asset questions used in the Kenyan Demographic Health Surveys	Principle component analysis (PCA) used to compute household SES: Lower/lower middle, middle, upper middle/upper
Proximal Socio-demographic factors		
Number of household dependants	“Not including yourself, how many people living in your household are dependent on your income?”	0, 1, 2 +
Recent hunger	Thinking now about the past 7 days, have you or anyone in your family skipped a meal because there was not enough food?	No vs Yes
Current social support	Do you have someone who you can talk to about your problems?	Yes/sometimes vs No

### Statistical analysis

Due to the skewed distribution of HCC levels, it was log-transformed for all statistical analyses. Descriptive statistics were conducted for each exposure variable and the outcome. Initial associations between HCC levels with the main exposure variables of interest and potential confounders were performed using univariate linear analyses with results in Tables 2 and 3, respectively. Violence, poor mental health, and harmful substance use variables, which were associated with HCC levels in univariate analyses (p < 0.1), were then taken forward for multivariable linear regression analyses adjusting for potential confounders that were associated (P < 0.1) with HCC levels in univariate analyses. In the multivariable regression models, we examined in separate models how violence (financial violence and physical and/or sexual violence), poor mental health (depression and PTSD), and harmful substance use (alcohol and/or other substances) were associated with HCC levels adjusted for confounders. To avoid multicollinearity and because of the high overlap among women who experienced different forms of violence, financial violence and physical and/ or sexual violence were placed in separate multivariable models. This logic was also applied to mental health problems (depression and PTSD) and harmful alcohol and substance use. To ascertain if the different exposures were independently associated with HCC levels, we created a model (Model 6) which included all significant (p < 0.05) exposure variables (from the previous models built) adjusted for confounders and each other. We then tested for a dose–response relationship by examining if an increasing number of exposure variables from Model 6 (i.e. no experience of recent physical and/or sexual violence and no harmful alcohol and /or other substance use vs. experience of recent physical and/or sexual violence OR harmful alcohol and /or other substance use vs. experience of recent physical and/or sexual violence AND harmful alcohol and /or other substance use) was associated with increasing HCC levels (Model 7). Finally, we examined whether HCC levels were associated with CRP levels using univariate linear regression with results in Table 3. CRP is sometimes used as a biomarker of acute systemic inflammation, with levels > 3ug/ml indicating adverse health risks, including active infection, coronary heart disease, and stress-related disorders [53, 54].

**Table 2:** Violence, mental health problems, and harmful alcohol and other substance use among study participants and their relationship with log-transformed HCC levels

	N (weighted %) (N = 425)	HCC Geometric mean ng/g^a^	P-value*
Recent violence			
Financial violence			0.022
No	148 (33.5)	274.56	
Yes	272 (66.5)	368.21	
Emotional violence			0.107
No	102 (23.0)	281.67	
Yes	323 (77.0)	347.59	
Physical violence			< 0.0001
No	195 (45.4)	265.7	
Yes	230 (54.6)	397.84	
Sexual violence			0.295
No	222 (50.6)	311.9	
Yes	203 (49.4)	352.21	
Physical and/or sexual violence			0.001
No	149 (33.6)	252.82	
Yes	276 (66.4)	379.78	
Any recent violence^b^			0.344
No	49(10.7)	285.63	
Yes	376 (89.3)	337.1	
Police arrest			0.995
No	302 (70.9)	331.28	
Yes	123 (29.1)	331.02	
Mental health problems			
Depression			0.041
None/Mild	329 (76.3)	310.53	
Moderate/Severe	95 (23.7)	411.81	
Anxiety			0.526
None/Mild	376 (88.4)	326.99	
Moderate/severe	49 (11.6)	365.06	
PTSD			0.013
Negative	365 (86.5)	312.43	
Positive	56 (13.5)	482.16	
Suicidal thoughts and/or attempt			0.949
No	379 (89.2)	331.68	
Yes	46 (10.8)	327.27	
Harmful substance use			
Alcohol use problem^c^			< 0.0001
Low risk	276 (65.7)	287.2	
Moderate/high risk	146 (34.3)	431.91	
Other substance use problem ^c,d^			0.015
Low risk	262 (63.5)	299.16	
Moderate/high risk	162 (36.5)	394.18	
Tobacco use			0.895
No	333 (78.5)	329.64	
Yes	92 (21.5)	336.98	
Alcohol and/or other substance use problem			0.001
No	212 (51.2)	277.28	
Yes	213 (48.8)	399.02	

^a^Geometric mean is the backward-transformed mean from the transformed data

^*^P-values were calculated using simple linear regression

^b^Refers to any recent financial, emotional, physical, or sexual violence

^c^Alcohol /other substance use problem: low risk 0–10; moderate/high risk 11 +

^d^Other substances (cannabis, cocaine, amphetamines, hallucinogens, sedatives, and inhalants) excluding tobacco smoking and alcohol

**Table 3:** Characteristics of study participants and associations with log-transformed HCC levels

Characteristic	N (weighted %) (N = 425)	HCC Geometric mean ng/g^a^	P-value*
Distal-demographic factors			
Age			0.640
< 25	119 (16.1)	325.1	
25–34	155 (42.5)	352.75	
35 +	151 (41.4)	312.71	
Age at first sex			0.10
< / = 15	130 (30.4)	361.78	
16–17	148 (33.8)	371.71	
18 +	143 (35.80)	279.5	
Literacy			0.507
Illiterate	61 (15.3)	360.96	
Literate	364 (84.7)	326.08	
Religion			0.373
Catholic	172 (39.7)	364.01	
Protestant	220 (52.8)	307.52	
Muslim/others/none	33 (7.5)	338.87	
Socio-economic status			0.081
Lower/lower middle	161 (37.2)	370.62	
Middle	76 (17.4)	380.56	
Upper middle/upper	188 (45.4)	286.45	
Total number of ACEs reported^b^			0.002
0 to 4	110 (25.4)	240.02	
5 to 8	246 (58.6)	355.84	
9 to 12	69 (16.0)	424.96	
Proximal-sociodemographic factors			
Marital Status			0.595
Single	125 (26.5)	346.55	
Married or cohabiting	28 (7.0)	250.16	
Separated/divorced /widowed	272 (66.5)	335	
Number of Children**			0.043
None	24 (4.8)	187.23	
1–2	274 (66.5)	366.69	
3 +	101 (28.6)	285.8	
Number of household dependents			0.739
0	82 (17.8)	332.81	
1	117 (25.4)	308.11	
2 +	226 (56.8)	341.57	
Recent Hunger			0.981
No	305 (70.8)	331.62	
Yes	119 (29.2)	330.62	
Have other source (s) of income			0.482
Yes	198 (47.6)	345.73	
No	227(52.4)	318.52	
Social support			0.204
No	119 (27.5)	370.27	
Yes	306 (72.5)	317.5	
Sex-work characteristics/sexual risk behaviours			
Place of selling sex			0.546
Lodge/hotel/rented room/home	406 (97.0)	336.09	
Public places	13 (3.1)	277.59	
Number of clients /weeks			0.888
< 5	246 (58.5)	337.7	
5 +	173 (41.6)	332.31	
Condom use last sex			0.044
Yes	313 (74.4)	311.19	
No	112 (25.6)	397.08	
Contraceptive use			0.702
No	61 (14.5)	349.71	
Yes	364 (85.5)	328.16	
Bacterial STI prevalence (Chlamydia/Gonorrhoea/syphilis)^b^			0.519
0	368 (87.6)	336.44	
1 +	57 (12.4)	296.59	
Experienced previous abortion/still birth**			0.793
No	222 (54.1)	325.7	
Yes	177 (45.9)	336.28	
Reports any sex-work related stigma			0.678
No	58 (13.0)	360.26	
Yes	361 (87.0)	333.17	
Systemic inflammation			
CRP Level (ug/ml)^c^			0.709
≤ 3	309 (71.9)	339.98	
> 3	111 (28.1)	323.19	

^a^Geometric mean is the backward-transformed mean from the transformed data

^*^P-values were calculated using simple linear regression

^**^Missing n = 26

^b^Bacterial STI prevalence: defined as a positive test for gonorrhoea, chlamydia and/or syphilis infection

^c^CRP level: None-low inflammation ≤ 3; high inflammation > 3

All analyses were weighted for age and adjusted for clustering by clinic. For easy interpretation, the mean of log-transformed HCC levels in descriptive analyses and the coefficients from linear regression models were exponentiated to produce geometric mean and geometric mean ratios (GMR), respectively [55]. The geometric mean ratio is the backward-transformed mean from the transformed data, while the GMR is the ratio of the means which have been backward-transformed [55]. All analyses were performed using STATA 16.1; statistical significance was set at p < 0.05, and variables with > 5% missing observations were reported.

## Results

425 HIV-negative women provided viable baseline hair samples. The prevalence of recent (past six months) violence of any form from an IP and/or non-IP was 89.3 (95% CI: 86.1–91.9) (physical 54.6%; sexual 49.4%; physical and/or sexual 66.4%; emotional 77% and financial 66.5%), and 29.1% had been arrested by the police in the past six months because of their sex work (Table 2). Mental health problems were common, with 23.7% reporting moderate/severe depression, 11.6% moderate/severe anxiety, 13.5% PTSD and 10.8% recent suicidal thoughts and/or attempts. About half of the participants had alcohol and/or substance use problems (48.8%) (alcohol use problems 34.3%; other substance use problems 36.5%), and about 21.5% had used tobacco in the past three months (Table 2).

The mean age of study participants was 33.7 years (age range: 18–45 years). Most (84.7%) were literate and Christian (Protestants 52.8%; Catholics 39.7%). The mean age of sexual debut was 16.3 years, with 30.4% of FSWs reporting first sex at 15 years or less. ACEs were commonly reported, with 89.8% reporting one or more ACEs. Relatively few (7.0%) were currently married or living with a sexual partner, while the majority (66.5%) had been divorced, widowed, or separated from their partners. Almost all participants (95.2%) had at least one child. Just under half of participants (47.6%) reported having other sources of income in addition to sex work, and 29.2% reported having missed a meal in the past seven days due to financial challenges. Most of the women sold sex in non-public places (97%), and 41.6% reported a client volume of at least 5 per week. The prevalence of reported condom use at last sex was 74.4%, and 12.4% had a bacterial sexually transmitted infection (STI). Sex-work-related stigma was common (87%)**,** and 28.1% had high CRP levels (> 3ug/ml) (Table 3).

Table 3 provides more details about participants' characteristics. When we compared the characteristics of participants in this study with those of the 321 HIV-negative participants who were excluded from this analysis due to insufficient hair samples, the participants were broadly similar (Supplementary Materials: Tables S1 and S2).

### Relationship of HCC levels with violence, mental health problems and harmful alcohol/substance use

The mean HCC was 331.20 (95% CI 294.7–372.2). In univariate analysis, recent financial violence, physical violence, physical and/or sexual violence, depression, harmful alcohol use, other substance use problems, and harmful alcohol and/or substance use were all associated with higher HCC (p < 0.05). In multivariable analyses (Table 4), there was no evidence of associations between either financial violence (adjusted GMR (aGMR) = 1.19; 95% CI 0.90–1.56) (Model 1) or depression (aGMR) = 1.24; 95% CI 0.92–1.66) (Model 3) and HCC levels, with weak evidence of an association between PTSD (aGMR) = 1.40; 95% CI 0.96–2.05) and HCC (Model 4). However, after adjusting for known confounders, we did find evidence that women who reported (i) recent physical and/or sexual violence (Model 2) and (ii) alcohol and/or other substance use problems (Model 5) had higher HCC levels compared to their unexposed counterparts When these last two interrelated exposures were included in the same model (Model 6), there was evidence that recent physical and/or sexual violence (aGMR) = 1.28; 95% CI 1.01–1.62) and recent alcohol and/or other substance use problems (aGMR = 1.31; 95% CI 1.03- to 1.65) were independently associated with increased HCC. Interestingly, we also found evidence of a dose–response with an increasing number of exposures associated with an increasing HCC level (Model 7).

**Table 4:** Multivariable linear regression of factors associated with log-transformed HCC levels

		Crude GMR (CI)	P-value	Adjusted GMR (CI)*	P-value
Model 1 (N = 391)	Financial Violence				
	No	Reference			
	Yes	1.34 (1.04–1.73)	0.022	1.19 (0.90–1.56)	0.219
Model 2 (N = 395)	Physical and/or Sexual Violence				
	No	Reference			
	Yes	1.50 (1.18–1.90)	0.001	1.29 (1.02–1.64)	0.034
Model 3 (N = 394)	Depression				
	No	Reference			
	Yes	1.33(1.01–1.74)	0.041	1.24 (0.92–1.66)	0.16
Model 4 (N = 391)	PTSD				
	No	Reference			
	Yes	1.54 (1.10–2.17)	0. 013	1.40 (0.96–2.05)	0.082
Model 5 (N = 395)	Alcohol and/or other substance use problem				
	No	Reference			
	Yes	1.44 (1.15–1.80)	0.001	1.32 (1.04–1.67)	0.02
Model 6 (N = 395)	Physical and/or Sexual Violence				
	No	Reference		Reference	
	Yes	1.50 (1.18–1.90)	0.001	1.28 (1.01–1.62)	0.044
	Alcohol and/or other substance use problem				
	No	Reference		Reference	
	Yes	1.44 (1.15–1.80)	0. 001	1.31 (1.03–1.65)	0.025
Model 7 (N = 395)	Exposure counts^a^				0.002^b^
	0	Reference		Reference	
	1	1.42 (1.04–1.95)		1.23 (0.89–1.70)	
	2	1.98 (1.44–2.71)	0.0001	1.65 (1.19–2.29)	0.007
	Socio-economic status				
	Lower/lower middle	Reference		Reference	
	middle	1.03 (0.78–1.36)		0.97 (0.72–1.30)	
	upper middle/upper	0.77 (0.60–0.99)	0.081	0.72 (0.54–0.95)	0.04
	Number of Children				
	0	Reference		Reference	
	1–2	1.95 (0.88–4.37)		2.24 (1.10–4.58)	
	3 +	1.53 (0.68–3.44)	0.043	1.55 (0.76–3.16)	0.004
	Condom use last sex				
	Yes	Reference		Reference	
	No	1.27 (1.01–1.62)	0.044	1.27 (1.00–1.63)	0.05

^*^Adjusted for age at first sex, socioeconomic status, total ACEs, number of children, condom use in last sex and SWOP clinic

^a^Exposures included the experience of physical and/or sexual violence and harmful alcohol and/or substance use (0 = no experience of recent physical and/or sexual violence and no harmful alcohol and/or other substance use; 1 = experience of recent physical and/or sexual violence OR harmful alcohol and/or other substance use; 2 = experience of recent physical and/or sexual violence AND harmful alcohol and/or other substance use)

^b^Test of linearity p-value

Potential confounding variables which showed evidence of association with HCC levels were higher socio-economic status levels (SES) (vs. lower SES level) (aGMR = 0.72; 95% CI 0.54–0.95), one or more children (vs. no children) (aGMR = 2.24; 95% CI 1.10–4.58) and non-condom use at last vaginal sex (aGMR 1.27; 95% CI 1.00–1.63).

### Relationship between HCC levels and CRP levels

When we examined the association between HCC and CRP levels, we found no evidence of an association in univariate regression (p = 0.709) (Table 3).

## Discussion

We found a high prevalence of recent violence experiences, common mental health problems and harmful alcohol and substance use among FSWs in Nairobi, Kenya. Moreover, we found evidence of positive and independent associations between HCC levels and (i) recent physical and/or sexual violence and (ii) alcohol and/or other substance use problems.

Several studies have examined violence against women in the general population and HCC [56]. However, most of those studies focused on a specific form of violence or combined all forms of violence (e.g., physical, sexual or emotional) into one variable. This makes it hard to fully comprehend how different forms of violence impact cortisol production. In addition, the paucity of studies with high-risk groups such as FSWs means it is not known how violence impacts cortisol production in different populations. In our research with FSWs, we found evidence that the recent experience of physical and/or sexual violence was associated with high HCC levels. This suggests a biological pathway linking the experience of recent violence and the stress-response system with an increase in HCC, which may continue over time before it is attenuated [37]. However, due to the limitation of cross-sectional studies in making causal inferences, longitudinal studies are needed to assess the direction of causality. Women who experienced physical and/or sexual violence have been reported to have a higher risk of HIV infection and mental health problems [43]. Although sexual violence as a separate variable was not found to be associated with HCC levels in univariate analysis, it is theoretically unlikely that physical violence, but not sexual violence, would increase HCC. We likely did not see associations between sexual violence and HCC levels, as our reference category for sexual violence included women who had experienced physical violence [22]. Our combined physical and/or sexual violence variable had a cleaner reference category and found evidence of an association with HCC. Although we could have examined associations between women who experienced sexual violence only without physical violence, and HCC levels the sample size was too small to have sufficient power to allow this. In practice, most women who experience sexual violence will also experience physical violence and thus it makes most sense to analyse this as a combined variable.

We found evidence of a positive association between HCC and alcohol and/or substance use problems, even after adjusting for physical and/or sexual violence. Our findings are consistent with the literature from other populations, suggesting that substance use, such as alcohol, may be associated with increased cortisol levels [57]. For example, a study of 23 alcoholics in the acute withdrawal phase and 25 abstinent alcoholics as controls found three to four-fold higher HCC among the alcoholics compared to the controls [36]. Cigarette or tobacco smoking was not associated with HCC in our study, which aligns with findings in the literature [57]. The survey conducted among FSWs in Mombasa, Kenya, found a similar lack of evidence between tobacco smoking and HCC [39]. Although some FSWs consume harmful substances as part of sex work or to cope with the stresses of sex work [4, 58], the same harmful substances may exacerbate poor mental health [26]. Even though participants in our study with depression, anxiety and PTSD had higher HCC levels in univariate analyses, the associations did not remain significant after adjusting for potential confounders. A study of FSWs in Mombasa, Kenya, found similar insignificant findings between HCC and depression and PTSD [39]. Mental health problems such as anxiety, depression and PTSD have been associated with HCC in other non-FSW populations, but findings have been inconsistent [57]. The heterogeneous findings may be attributed to differences in the characteristics of study populations [59].

Findings in this study suggest a role of violence and substance use in elevated HCC levels. Increased HCC has been associated with a range of health conditions, including cardiovascular diseases, diabetes mellitus, metabolic diseases, and immune function impairment [26]. Although our study found no evidence of an association between HCC and CRP levels, cortisol is known to play a key role in regulating the immune system, including T-cell activation. Hence, increased HCC levels may contribute to an increased likelihood of acquiring HIV in this population at high risk for HIV [26]. However, prospective longitudinal studies and mechanistic studies that measure the incidence of HIV infection, susceptibility, and HIV-relevant immune function are needed to test this hypothesis in this vulnerable population.

### Strengths and limitations

A strength of our study is the large random sample of FSWs included and the use of validated tools to measure violence, mental health problems, alcohol and substance use problems and HCC. Limitations include the cross-sectional study design, so we can neither assume a temporal relationship nor infer causality, as well as the useability of only around 56% of hair samples provided due to extremely short hair or hair samples with too few strands. Our focus on women with useable 2 cm of hair meant the hair sample time frame (past 2.5 months) may not have matched the violence exposure timeframes (past six months) or the mental health (past two weeks) time frames. Additionally, CRP is a non-specific marker of acute inflammation [60], so it could pick up any infections that participants experienced on the day of blood sampling (e.g., flu) but might miss inflammation that might have occurred weeks prior. This could potentially impact the ability to detect an association between HCC and CRP levels if such an association exists. Another possible bias could be that the characteristics of participants in this study may differ from the overall HIV-negative and the baseline participants of the *Maisha Fiti* study in general. However, this bias may be minimal as the prevalence of our main exposure variables and other characteristics of study participants in this study are similar to that of the overall HIV-negative participants (Supplementary Materials: Tables S1 and S2) and the baseline cohort of the *Maisha Fiti* study published elsewhere [8, 9, 11]. Furthermore, our exposure measures were retrospective, with the possibility of recall bias. Nonetheless, studies have revealed a moderate concordance between prospective and retrospective reporting of similar measures [61, 62]. Also, this study may be prone to underreporting sensitive issues, including our exposure measures, meaning the prevalence we reported may be underestimated. In addition, we did aim to include all known confounders from the literature to adjust for our analyses; however, it is plausible that we missed important confounders. Lastly, there is a need for prospective and retrospective research on cortisol, especially in defining the normal reference range in different populations.

## Conclusion

In conclusion, among FSWs in Nairobi, we find independent positive associations between HCC levels and recent physical and/or sexual violence and alcohol and/or substance use problems, with a cumulative dose–response relationship seen with increasing exposures. This suggests that increased HCC due to violence and harmful substances could be associated with increased HIV-1 acquisition risk; however, there is a need for more research to understand this further. Moreover, future studies could investigate how other more relevant biomarkers of the inflammatory systemic response system are associated with violence, harmful alcohol/substance use and HCC in this population. Together, this will be useful in understanding how FSWs’ experiences of violence, poor mental health and harmful alcohol/substance use may increase their risk of HIV through biological pathways.

### Supplementary Information

Below is the link to the electronic supplementary material.

Supplementary file 1 (DOCX 31 KB)

## Data Availability

The datasets generated during and/or analyzed during the current study are available from the corresponding author upon reasonable request.

## References

[1] World Health Organisation. Violence against women prevalence estimates, 2018; global, regional and national prevalence estimates for intimate partner violence against women and global and regional prevalence estimates for non-partner sexual violence against women. Geneva: WHO; 2018.

[2] Scorgie F, Chersich MF, Ntaganira I, Gerbase A, Lule F, Lo YR. Socio-demographic characteristics and behavioral risk factors of female sex workers in sub-saharan Africa: a systematic review. AIDS Behav. 2012;16(4):920–33. 21750918 10.1007/s10461-011-9985-z

[3] Martín-Romo L, Sanmartín FJ, Velasco J. Invisible and stigmatized: a systematic review of mental health and risk factors among sex workers. Acta Psychiatr Scand. 2023. 10.1111/acps.13559. 37105542 10.1111/acps.13559

[4] Mbonye M, Nakamanya S, Nalukenge W, King R, Vandepitte J, Seeley J. ‘It is like a tomato stall where someone can pick what he likes’: structure and practices of female sex work in Kampala, Uganda. BMC Public Health. 2013;13(1):741. 23938037 10.1186/1471-2458-13-741 PMC3751244

[5] Joint United Nations Programme on HIV/AIDS. Confronting inequalities: lessons for pandemic responses from 40 years of AIDS. Global AIDS update 2021.

[6] Joint United Nations Programme on HIV/AIDS. HIV and sex work—Human rights fact sheet series. 2021. https://www.unaids.org/en/resources/documents/2021/05-hiv-human-rights-factsheet-sex-work. Accessed 24 Jun 2023.

[7] Panneh M, Gafos M, Nyariki E, Liku J, Shah P, Wanjiru R, Wanjiru M, Beksinska A, Pollock J, Gwala D, et al. Mental health challenges and perceived risks among female sex workers in Nairobi, Kenya. BMC Public Health. 2022;22(1):2158. 36418973 10.1186/s12889-022-14527-5 PMC9685887

[8] Beksinska A, Nyariki E, Kabuti R, Kungu M, Babu H, Shah P, Nyabuto C, Okumu M, Mahero A, et al. Harmful alcohol and drug use is associated with syndemic risk factors among female sex workers in Nairobi, Kenya. Int J Environ Res Public Health. 2022. 10.3390/ijerph19127294. 35742558 10.3390/ijerph19127294 PMC9223659

[9] Beksinska A, Jama Z, Kabuti R, Kungu M, Babu H, Nyariki E, Shah P, Nyabuto C, Okumu M, Maisha Fiti Study C, et al. Prevalence and correlates of common mental health problems and recent suicidal thoughts and behaviours among female sex workers in Nairobi, Kenya. BMC Psychiatry. 2021;21(1):503–503. 34649544 10.1186/s12888-021-03515-5 PMC8518166

[10] Agot K, Cain M, Medley A, Kimani J, Gichangi P, Kiio C, Mukiri E, Odonde P, Toroitich-Ruto C, Bingham T, et al. Formative assessment to identify perceived benefits and barriers of HIV oral self-testing among female sex workers, service providers, outreach workers, and peer educators to inform scale-up in Kenya. AIDS Care. 2022;34(6):717–24. 33657929 10.1080/09540121.2021.1894318 PMC10962321

[11] Beattie TS, Kabuti R, Beksinska A, Babu H, Kung’u M, Shah P, Nyariki E, Nyamweya C, Okumu M, The Maisha Fiti Study C, et al. Violence across the life course and implications for intervention design: findings from the maisha fiti study with female sex workers in Nairobi, Kenya. Int J Environ Res Public Health. 2023. 10.3390/ijerph20116046. 37297650 10.3390/ijerph20116046 PMC10253020

[12] Kenya Legal & Ethical Issues Network. Punitive laws affecting sex. 2016. https://www.kelinkenya.org/wp-content/uploads/2016/03/PUNITIVE-LAWS-AFFECTING-SEX-WORKERS.pdf. Accessed 15 Mar 2024.

[13] Mbote DK, Nyblade L, Kemunto C, Giger K, Kimani J, Mingkwan P, Njuguna S, Oga E, Kraemer JD. Police discrimination, misconduct, and stigmatization of female sex workers in Kenya: associations with delayed and avoided health care utilization and lower consistent condom use. Health Hum Rights. 2020;22(2):199–212. 33390707 PMC7762893

[14] Devries KM, Mak JY, Bacchus LJ, Child JC, Falder G, Petzold M, Astbury J, Watts CH. Intimate partner violence and incident depressive symptoms and suicide attempts: a systematic review of longitudinal studies. PLoS Med. 2013;10(5):e1001439. 23671407 10.1371/journal.pmed.1001439 PMC3646718

[15] Devries KM, Child JC, Bacchus LJ, Mak J, Falder G, Graham K, Watts C, Heise L. Intimate partner violence victimization and alcohol consumption in women: a systematic review and meta-analysis. Addiction. 2014;109(3):379–91. 24329907 10.1111/add.12393

[16] Pacek LR, Martins SS, Crum RM. The bidirectional relationships between alcohol, cannabis, co-occurring alcohol and cannabis use disorders with major depressive disorder: results from a national sample. J Affect Disord. 2013;148(2–3):188–95. 23260381 10.1016/j.jad.2012.11.059 PMC3608823

[17] Li Y, Marshall CM, Rees HC, Nunez A, Ezeanolue EE, Ehiri JE. Intimate partner violence and HIV infection among women: a systematic review and meta-analysis. J Int AIDS Soc. 2014;17(1):18845. 24560342 10.7448/IAS.17.1.18845 PMC3925800

[18] Remien RH, Stirratt MJ, Nguyen N, Robbins RN, Pala AN, Mellins CA. Mental health and HIV/AIDS: the need for an integrated response. AIDS. 2019;33(9):1411–20. 30950883 10.1097/QAD.0000000000002227 PMC6635049

[19] Rehm J, Probst C, Shield KD, Shuper PA. Does alcohol use have a causal effect on HIV incidence and disease progression? A review of the literature and a modeling strategy for quantifying the effect. Popul Health Metrics. 2017;15(1):4. 10.1186/s12963-017-0121-9 PMC5301358 28183309

[20] Huff HV, Carcamo PM, Diaz MM, Conklin JL, Salvatierra J, Aponte R, Garcia PJ. HIV and substance use in Latin America: a scoping review. Int J Environ Res Public Health. 2022. 10.3390/ijerph19127198. 35742448 10.3390/ijerph19127198 PMC9222977

[21] Tsuyuki K, Cimino AN, Holliday CN, Campbell JC, Al-Alusi NA, Stockman JK. Physiological changes from violence-induced stress and trauma enhance HIV susceptibility among women. Curr HIV/AIDS Rep. 2019;16(1):57–65. 30762216 10.1007/s11904-019-00435-8 PMC6420839

[22] Durevall D, Lindskog A. Intimate partner violence and HIV in ten sub-Saharan African countries: what do the demographic and health surveys tell us? Lancet Glob Health. 2015;3(1):e34-43. 25539967 10.1016/S2214-109X(14)70343-2

[23] Kalokhe AS, Ibegbu CC, Kaur SP, Amara RR, Kelley ME, Del Rio C, et al. Intimate partner violence is associated with increased cd4(+) t-cell activation among HIV-negative high-risk women. Pathog Immun. 2016;1(1):193–213. 10.20411/pai.v1i1.120. 27668294 10.20411/pai.v1i1.120 PMC5034930

[24] Segerstrom SC, Miller GE. Psychological stress and the human immune system: a meta-analytic study of 30 years of inquiry. Psychol Bull. 2004;130(4):601–30. 15250815 10.1037/0033-2909.130.4.601 PMC1361287

[25] Aiyer SM, Heinze JE, Miller AL, Stoddard SA, Zimmerman MA. Exposure to violence predicting cortisol response during adolescence and early adulthood: understanding moderating factors. J Youth Adolesc. 2014;43(7):1066–79. 24458765 10.1007/s10964-014-0097-8 PMC4057303

[26] Jones C, Gwenin C. Cortisol level dysregulation and its prevalence-Is it nature’s alarm clock? Physiol Rep. 2021;8(24):e14644. 33340273 10.14814/phy2.14644 PMC7749606

[27] Cohen S, Janicki-Deverts D, Doyle WJ, Miller GE, Frank E, Rabin BS, Turner RB. Chronic stress, glucocorticoid receptor resistance, inflammation, and disease risk. Proc Natl Acad Sci U S A. 2012;109(16):5995–9. 22474371 10.1073/pnas.1118355109 PMC3341031

[28] Patterson S, Moran P, Epel E, Sinclair E, Kemeny ME, Deeks SG, Bacchetti P, Acree M, Epling L, Kirschbaum C, et al. Cortisol patterns are associated with T cell activation in HIV. PLoS ONE. 2013;8(7):e63429. 23922644 10.1371/journal.pone.0063429 PMC3724863

[29] Passmore J-AS, Jaspan HB, Masson L. Genital inflammation, immune activation and risk of sexual HIV acquisition. Curr Opinion HIV AIDS. 2016;11(2):156–62. 10.1097/COH.0000000000000232 PMC6194860 26628324

[30] Heinze K, Lin A, Reniers R, Wood SJ. Longer-term increased cortisol levels in young people with mental health problems. Psychiatry Res. 2016;236:98–104. 26749569 10.1016/j.psychres.2015.12.025 PMC4756272

[31] Dziurkowska E, Wesolowski M. Cortisol as a biomarker of mental disorder severity. J Clin Med. 2021. 10.3390/jcm10215204. 34768724 10.3390/jcm10215204 PMC8584322

[32] Carpenter LL, Tyrka AR, Ross NS, Khoury L, Anderson GM, Price LH. Effect of childhood emotional abuse and age on cortisol responsivity in adulthood. Biol Psychiatry. 2009;66(1):69–75. 19375070 10.1016/j.biopsych.2009.02.030 PMC2696583

[33] Peckins MK, Roberts AG, Hein TC, Hyde LW, Mitchell C, Brooks-Gunn J, McLanahan SS, Monk CS, Lopez-Duran NL. Violence exposure and social deprivation is associated with cortisol reactivity in urban adolescents. Psychoneuroendocrinology. 2020;111:104426. 31639588 10.1016/j.psyneuen.2019.104426 PMC7266108

[34] Staufenbiel SM, Penninx BW, Spijker AT, Elzinga BM, van Rossum EF. Hair cortisol, stress exposure, and mental health in humans: a systematic review. Psychoneuroendocrinology. 2013;38(8):1220–35. 23253896 10.1016/j.psyneuen.2012.11.015

[35] Chen K, Hollunder B, Garbusow M, Sebold M, Heinz A. The physiological responses to acute stress in alcohol-dependent patients: a systematic review. Eur Neuropsychopharmacol. 2020;41:1–15. 32994116 10.1016/j.euroneuro.2020.09.003

[36] Stalder T, Kirschbaum C, Heinze K, Steudte S, Foley P, Tietze A, Dettenborn L. Use of hair cortisol analysis to detect hypercortisolism during active drinking phases in alcohol-dependent individuals. Biol Psychol. 2010;85(3):357–60. 20727937 10.1016/j.biopsycho.2010.08.005

[37] Steudte-Schmiedgen S, Kirschbaum C, Alexander N, Stalder T. An integrative model linking traumatization, cortisol dysregulation and posttraumatic stress disorder: Insight from recent hair cortisol findings. Neurosci Biobehav Rev. 2016;69:124–35. 27443960 10.1016/j.neubiorev.2016.07.015

[38] Alhalal E, Falatah R. Intimate partner violence and hair cortisol concentration: a biomarker for HPA axis function. Psychoneuroendocrinology. 2020;122:104897. 33068953 10.1016/j.psyneuen.2020.104897

[39] Heller M, Roberts ST, Masese L, Ngina J, Chohan N, Chohan V, Shafi J, McClelland RS, Brindle E, Graham SM. Gender-based violence, physiological stress, and inflammation: a cross-sectional study. J Womens Health (Larchmt). 2018;27(9):1152–61. 29630431 10.1089/jwh.2017.6743 PMC6148727

[40] Greff MJE, Levine JM, Abuzgaia AM, Elzagallaai AA, Rieder MJ, van Uum SHM. Hair cortisol analysis: an update on methodological considerations and clinical applications. Clin Biochem. 2019;63:1–9. 30261181 10.1016/j.clinbiochem.2018.09.010

[41] World Health Organization. WHO multi-country study on women's health and domestic violence against women: initial results on prevalence, health outcomes and women's responses. 2005. https://apps.who.int/iris/handle/10665/43309. Accessed 19 Jun 2023.

[42] El-Bassel N, Mukherjee TI, Stoicescu C, Starbird LE, Stockman JK, Frye V, Gilbert L. Intertwined epidemics: progress, gaps, and opportunities to address intimate partner violence and HIV among key populations of women. Lancet HIV. 2022;9(3):e202–13. 35151376 10.1016/S2352-3018(21)00325-8 PMC10009883

[43] Devries KM, Mak JYT, García-Moreno C, Petzold M, Child JC, Falder G, Lim S, Bacchus LJ, Engell RE, Rosenfeld L, et al. The global prevalence of intimate partner violence against women. Science. 2013;340:1527–8. 23788730 10.1126/science.1240937

[44] Jewkes R, Fulu E, Tabassam Naved R, Chirwa E, Dunkle K, Haardörfer R, Garcia-Moreno C. Women’s and men’s reports of past-year prevalence of intimate partner violence and rape and women’s risk factors for intimate partner violence: a multicountry cross-sectional study in Asia and the Pacific. PLoS Med. 2017;14(9):e1002381. 28873087 10.1371/journal.pmed.1002381 PMC5584751

[45] Kroenke K, Spitzer RL, Williams JB. The PHQ-9: validity of a brief depression severity measure. J Gen Intern Med. 2001;16(9):606–13. 11556941 10.1046/j.1525-1497.2001.016009606.x PMC1495268

[46] Spitzer RL, Kroenke K, Williams JB, Löwe B. A brief measure for assessing generalized anxiety disorder: the GAD-7. Arch Intern Med. 2006;166(10):1092–7. 16717171 10.1001/archinte.166.10.1092

[47] Hossain M, Zimmerman C, Abas M, Light M, Watts C. The relationship of trauma to mental disorders among trafficked and sexually exploited girls and women. Am J Public Health. 2010;100(12):2442–9. 20966379 10.2105/AJPH.2009.173229 PMC2978168

[48] American Psychiatric Association. Diagnostic and statistical manual of mental disorders. 4th ed. Washington DC: American Psychiatric Association; 2000.

[49] McHugh CM, Corderoy A, Ryan CJ, Hickie IB, Large MM. Association between suicidal ideation and suicide: meta-analyses of odds ratios, sensitivity, specificity and positive predictive value. BJPsych Open. 2019;5(2):e18. 30702058 10.1192/bjo.2018.88 PMC6401538

[50] World Health Organisation. Preventing suicide: a global imperative. Geneva: World Health Organisation; 2014.

[51] World Health Organization. The alcohol, smoking and substance involvement screening test (ASSIST) manual for use in primary care. Geneva: WHO; 2010.

[52] Krieger N. Methods for the scientific study of discrimination and health: an ecosocial approach. Am J Public Health. 2012;102(5):936–44. 22420803 10.2105/AJPH.2011.300544 PMC3484783

[53] Kennedy E, Niedzwiedz CL. The association of anxiety and stress-related disorders with C-reactive protein (CRP) within UK Biobank. Brain Behavior Immunity Health. 2022;19:100410. 35028602 10.1016/j.bbih.2021.100410 PMC8741412

[54] Shah T, Casas JP, Cooper JA, Tzoulaki I, Sofat R, McCormack V, Smeeth L, Deanfield JE, Lowe GD, Rumley A, et al. Critical appraisal of CRP measurement for the prediction of coronary heart disease events: new data and systematic review of 31 prospective cohorts. Int J Epidemiol. 2008;38(1):217–31. 18930961 10.1093/ije/dyn217 PMC2639366

[55] Lee DK. Data transformation: a focus on the interpretation. Korean J Anesthesiol. 2020;73(6):503–8. 33271009 10.4097/kja.20137 PMC7714623

[56] Lynch R, Aspelund T, Kormáksson M, Flores-Torres MH, Hauksdóttir A, Arnberg FK, Lajous M, Kirschbaum C, Valdimarsdóttir U. Lifetime exposure to violence and other life stressors and hair cortisol concentration in women. Stress. 2022;25(1):48–56. 34962229 10.1080/10253890.2021.2011204

[57] Wosu AC, Valdimarsdóttir U, Shields AE, Williams DR, Williams MA. Correlates of cortisol in human hair: implications for epidemiologic studies on health effects of chronic stress. Ann Epidemiol. 2013;23(12):797-811.e792. 24184029 10.1016/j.annepidem.2013.09.006 PMC3963409

[58] Spencer RL, Hutchison KE. Alcohol, aging, and the stress response. Alcohol Res Health. 1999;23(4):272–83. 10890824 PMC6760387

[59] Steudte S, Kolassa I-T, Stalder T, Pfeiffer A, Kirschbaum C, Elbert T. Increased cortisol concentrations in hair of severely traumatized Ugandan individuals with PTSD. Psychoneuroendocrinology. 2011;36(8):1193–200. 21411229 10.1016/j.psyneuen.2011.02.012

[60] Sproston NR, Ashworth JJ. Role of C-reactive protein at sites of inflammation and infection. Front Immunol. 2018;9:754. 29706967 10.3389/fimmu.2018.00754 PMC5908901

[61] Colman I, Kingsbury M, Garad Y, Zeng Y, Naicker K, Patten S, Jones PB, Wild TC, Thompson AH. Consistency in adult reporting of adverse childhood experiences. Psychol Med. 2016;46(3):543–9. 26511669 10.1017/S0033291715002032

[62] Reuben A, Moffitt TE, Caspi A, Belsky DW, Harrington H, Schroeder F, Hogan S, Ramrakha S, Poulton R, Danese A. Lest we forget: comparing retrospective and prospective assessments of adverse childhood experiences in the prediction of adult health. J Child Psychol Psychiatry. 2016;57(10):1103–12. 27647050 10.1111/jcpp.12621 PMC5234278

