# Supplementary material for: Associations of hair cortisol levels with violence, poor mental health, and harmful alcohol and other substance use among female sex workers in Nairobi, Kenya
Source: Discov Ment Health. 2024 Aug 28;4(1):29. doi: 10.1007/s44192-024-00086-1 (PMC11358571; doi:10.1007/s44192-024-00086-1)
Supplement: Supplementary file 1 — (DOCX 31 KB) [file 44192_2024_86_MOESM1_ESM.docx]

**Associations of hair cortisol levels with violence, poor mental health, and harmful alcohol and other substance use among female sex workers in Nairobi, Kenya**

Mamtuti Panneh, MMSc^1^*, Qingming Ding, PhD^2^, Rhoda Kabuti, MSc^3^, The Maisha Fiti study champions^3^, John Bradley, PhD^4^, Polly Ngurukiri, BA^3^, Mary Kungu, BA^3^, Tanya Abramsky, MSc^1^, James Pollock, BScH^5^, Alicja Beksinska, BMBS^1^, Pooja Shah, MSc^1^, Erastus Irungu, MSc^3^, Mitzy Gafos, PhD^1^, Janet Seeley, PhD^1^, Helen A Weiss, DPhil^4^, Abdelbaset A. Elzagallaai, PhD^2^, Michael J Rieder, PhD^2^, Rupert Kaul, PhD^6^, Joshua Kimani, MBChB^3^**, Tara Beattie, PhD^1^**

^1^Department for Global Health and Development, London School of Hygiene & Tropical Medicine, London, UK. Mamtuti.Panneh@lshtm.ac.uk.

^2^Robarts Research Institute, Schulich School of Medicine and Dentistry, Western University, London, ON, Canada.

^3^Partners for Health and Development in Africa, Nairobi, Kenya.

^4^MRC International Statistics and Epidemiology Group, Department for Infectious Disease Epidemiology, LSHTM, London, UK

^5^Department of Immunology, University of Toronto, Toronto, Canada

^6^Department of Medicine, University of Toronto, Toronto, Canada.

*Corresponding author: Email [Mamtuti.panneh@lshtm.ac.uk](mailto:Mamtuti.panneh@lshtm.ac.uk)

** Authors contributed equally

Discover Mental Health Journal

| **Table S1.** Comparison of the prevalence of violence, mental health, and substance use disorder among study participants (N=425) and the HIV-negative participants who were excluded in the study (N=321) |
| --- |
|  |

|  | Total 425  N (%) | Total 321  N (%) |
| --- | --- | --- |
|  |  |  |
| Financial violence |  |  |
| No | 148 (33.5) | 102 (32.3) |
| Yes | 272 (66.5) | 218 (67.7) |
| Emotional violence |  |  |
| No | 102 (23.0) | 85(26.4) |
| Yes | 323 (77.0) | 236(73.6) |
| Physical violence |  |  |
| No | 195 (45.4) | 147 (43.1) |
| Yes | 230 (54.6) | 184 (56.86) |
| Sexual violence |  |  |
| No | 222 (50.6) | 162 (50.1) |
| Yes | 203 (49.4) | 159 (49.9) |
| Physical and / or sexual violence |  |  |
| No | 149 (33.6) | 110 (34.4) |
| Yes | 276 (66.4) | 211 (65.6) |
| Any recent violence^a^ |  |  |
| No | 49(10.7) | 42 (13.2) |
| Yes | 376 (89.3) | 279 (86.8) |
| Police arrest |  |  |
| No | 302 (70.9) | 226 (68.6) |
| Yes | 123 (29.1) | 95 (31.4) |
| Depression |  |  |
| None/Mild | 329 (76.3) | 252 (76.8) |
| Moderate/Severe | 95 (23.7) | 69 (23.2) |
| Anxiety |  |  |
| None/Mild | 376 (88.4) | 294 (91.4) |
| Moderate/severe | 49 (11.6) | 27 (8.6) |
| PTSD |  |  |
| Negative | 365 (86.5) | 272 (84.9) |
| Positive | 56 (13.5) | 46 (14.1) |
| Suicidal behaviours |  |  |
| No | 379 (89.2) | 292 (90.6) |
| Yes | 46 (10.8) | 29 (9.5) |
| Alcohol use problem^b^ |  |  |
| low risk | 276 (65.7) | 210 (65.5) |
| moderate/high risk | 146 (34.3) | 110 (34.5) |
| Other substance use problem ^b,c^ |  |  |
| low risk | 262 (63.5) | 213 (67.5) |
| moderate/high risk | 162 (36.5) | 107 (32.6) |
| Tobacco use |  |  |
| No | 333 (78.5) | 260 (80.8) |
| Yes | 92 (21.5) | 61 (19.3) |
| Alcohol and/or other substance use problem |  |  |
| No | 212 (51.2) | 160 (50.63) |
| Yes | 213 (48.8) | 161 (49.4) |

| ^a^refers to any recent financial, emotional, physical, or sexual violence  ^b^ alcohol /other substance use problem: low risk 0-10 moderate/high risk 11+ |
| --- |
| ^c^other substances (cannabis, cocaine, amphetamines, hallucinogens, sedatives and inhalant) excluding tobacco smoking and alcohol. |

**Table S2.** Comparison of the characteristics of study participants (N=425) and the HIV-negative participants who were excluded in the study (N=321)

| Characteristic | Total 425  N (%) | Total 321  N (%) |
| --- | --- | --- |
| Age |  |  |
| <25 | 119 (16.1) | 81 (14.3) |
| 25-34 | 155 (42.5) | 131 (46.8) |
| 35+ | 151 (41.4) | 109 (38.9) |
| Age at first sex |  |  |
| </=15 | 130 (30.4) | 122 (39.0) |
| 16-17 | 148 (33.8) | 88 (25.5) |
| 18+ | 143 (35.80) | 108 (35.5) |
| Literacy |  |  |
| illiterate | 61 (15.3) | 49 (16.59) |
| literate | 364 (84.7) | 272 (83.41) |
| Religion |  |  |
| Catholic | 172 (39.7) | 114 (35.2) |
| Protestant | 220 (52.8) | 165 (53.2) |
| Muslim/others/none | 33 (7.5) | 40 (11.6) |
| Socio-economic status |  |  |
| Lower/lower middle | 161 (37.2) | 138 (41.9) |
| middle | 76 (17.4) | 61 (18.9) |
| upper middle/upper | 188 (45.4) | 122 (39.2) |
| Total number of ACEs reported |  |  |
| 0 to 4 | 110 (25.4) | 86 (27.6) |
| 5 to 8 | 246 (58.6) | 182 (56.0) |
| 9 to 12 | 69 (16.0) | 53 (16.4) |
| Marital Status |  |  |
| Single | 125 (26.5) | 94 (26.2) |
| Married or cohabiting | 28 (7.0) | 29 (9.1) |
| Separated/divorced /widowed | 272 (66.5) | 198 (64.8) |
| Number of Children* |  |  |
| None | 24 (4.8) | 22 (2.8) |
| one to two | 274 (66.5) | 216 (69.8) |
| 3+ | 101 (28.6) | 74 (27.5) |
| Number of household dependents |  |  |
| 0 | 82 (17.8) | 58 (16.7) |
| 1 | 117 (25.4) | 72 (21.2) |
| 2+ | 226 (56.8) | 191 (62.1) |
| Recent Hunger |  |  |
| No | 305 (70.8) | 206 (63.7) |
| Yes | 119 (29.2) | 114 (36.3) |
| Have other source (s) of income |  |  |
| Yes | 198 (47.6) | 130 (40.5) |
| No | 227(52.4) | 191 (59.5) |
| Social support |  |  |
| No | 119 (27.5) | 88 (28.0) |
| Yes | 306 (72.5) | 233 (72.0) |
| Place of selling sex |  |  |
| Lodge/hotel/rented room/home | 406 (97.0) | 312 (97.2) |
| Public places | 13 (3.1) | 9 (2.9) |
| Number of clients /weeks |  |  |
| <5 | 246 (58.5) | 190 (58.5) |
| 5+ | 173 (41.6) | 131 (41.5) |
| Condom use last sex |  |  |
| yes | 313 (74.4) | 246 (77.7) |
| No | 112 (25.6) | 74 (22.3) |
| Contraceptive use |  |  |
| No | 61 (14.5) | 53 (16.4) |
| Yes | 364 (85.5) | 268 (83.6) |
| Bacterial STI prevalence (Chlamydia/Gonorrhoea/syphilis) ^a^ |  |  |
| none | 368 (87.6) | 284 (89.7) |
| One+ | 57 (12.4) | 37 (10.3) |
| Experienced previous abortion/still birth* |  |  |
| No | 222 (54.1) | 186 (60.2) |
| Yes | 177 (45.9) | 116 (39.8) |
| Reports any sex-work related stigma |  |  |
| No | 58 (13.0) | 46 (15.0) |
| Yes | 361 (87.0) | 274 (85.0) |

| *Missing n=26 |
| --- |

^a^Bacterial STI prevalence is defined as a positive test for gonorrhoea, chlamydia and/or syphilis infection.
